# Supplementary material for: Antimycobacterial and Anticancer Properties of Myrtus communis Leaf Extract
Source: Pharmaceuticals (Basel). 2024 Jul 2;17(7):872. doi: 10.3390/ph17070872 (PMC11279575; doi:10.3390/ph17070872)
Supplement: Supplementary file 1 [file pharmaceuticals-17-00872-s001.zip › pharmaceuticals-3003337-supplementary/Pharmaceuticals sup figures/Sup. Fig. S1.pdf]

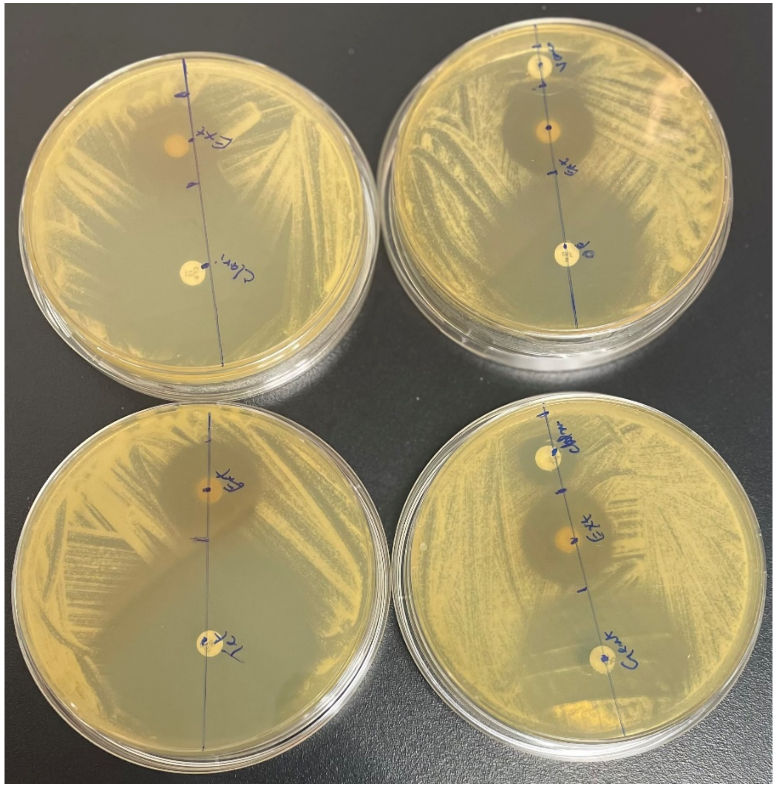

Supplementary Figure S1. Double disc synergy test of the extract with tetracyclin, gentamycin, chloramphenicol, ofloxacin, clarithromycin, and vancomycin against M. smegmatis
