# Supplementary material for: Antimycobacterial and Anticancer Properties of Myrtus communis Leaf Extract
Source: Pharmaceuticals (Basel). 2024 Jul 2;17(7):872. doi: 10.3390/ph17070872 (PMC11279575; doi:10.3390/ph17070872)
Supplement: Supplementary file 1 [file pharmaceuticals-17-00872-s001.zip › pharmaceuticals-3003337-supplementary/Pharmaceuticals sup figures/Sup. Fig. S3.pdf]

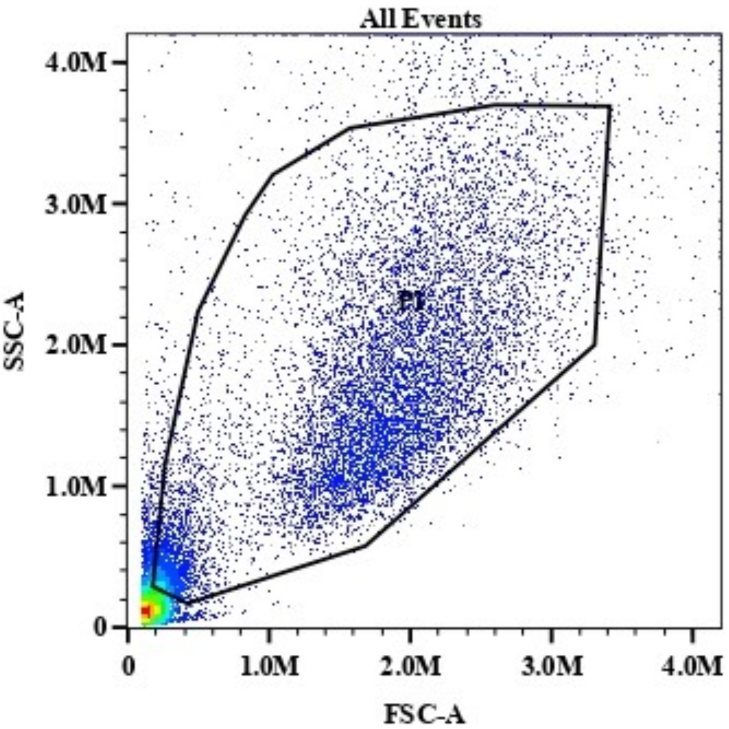


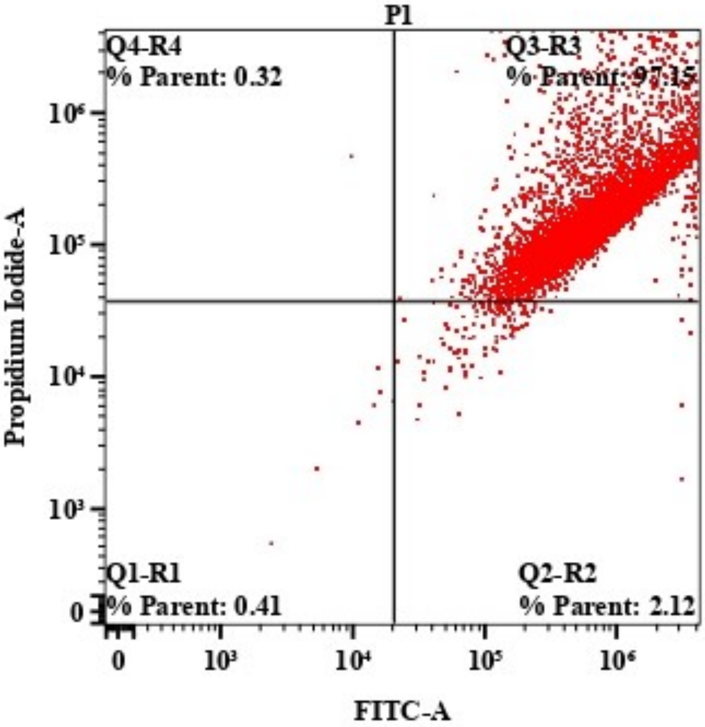


**Population Hierarchy**
**Dr Moshtaaq-MCF-7-PLANT EXTRACTION**

| Population | % Parent | Count |
|---|---|---|
| All Events | 100.00 | 21,536 |
| P1 | 51.94 | 11,186 |
| Q4-R4 | 0.32 | 36 |
| Q3-R3 | 97.15 | 10,867 |
| Q1-R1 | 0.41 | 46 |
| Q2-R2 | 2.12 | 237 |

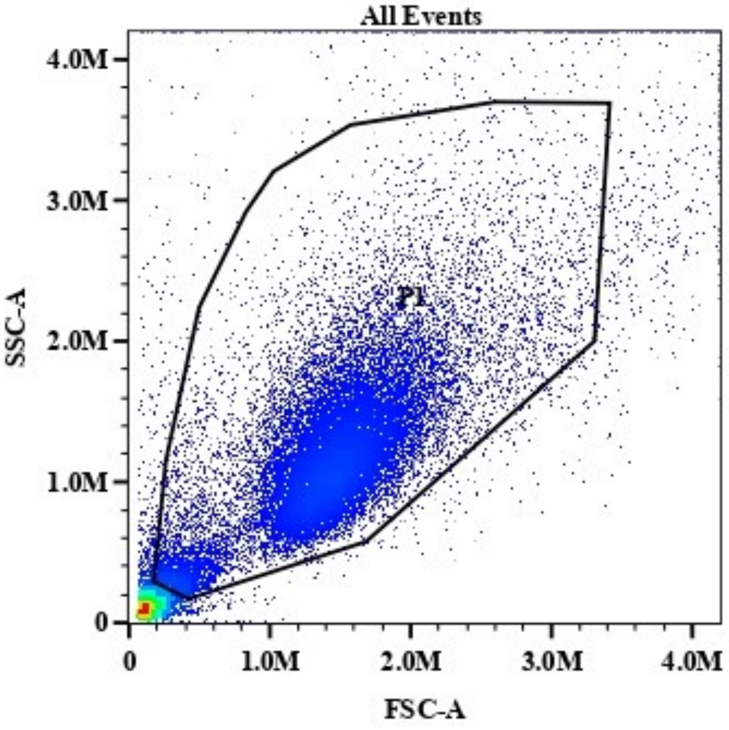


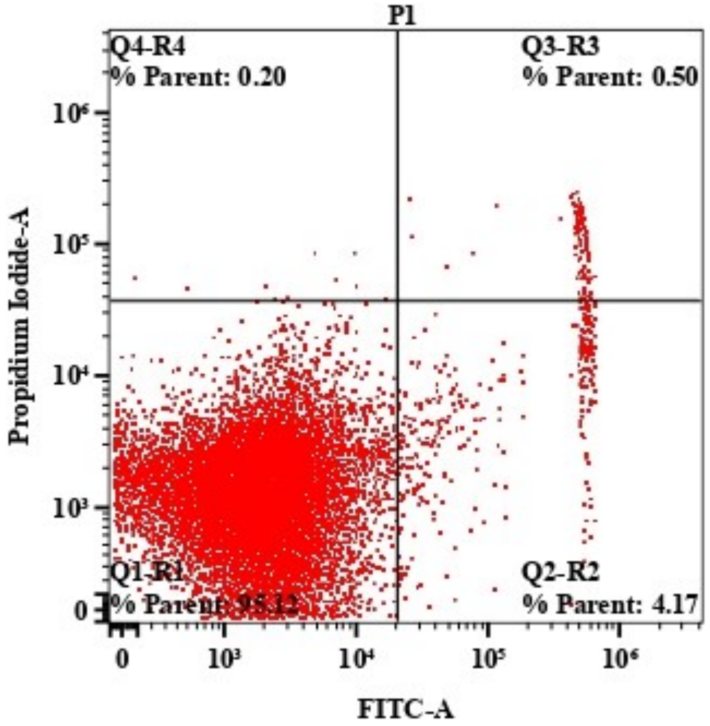


**Population Hierarchy**
**Dr Moshtaaq-MCF-7-MCF-7 CONTROL**

| Population | % Parent | Count |
|---|---|---|
| All Events | 100.00 | 50,000 |
| P1 | 52.14 | 26,071 |
| Q4-R4 | 0.20 | 53 |
| Q3-R3 | 0.50 | 131 |
| Q1-R1 | 95.12 | 24,799 |
| Q2-R2 | 4.17 | 1,088 |

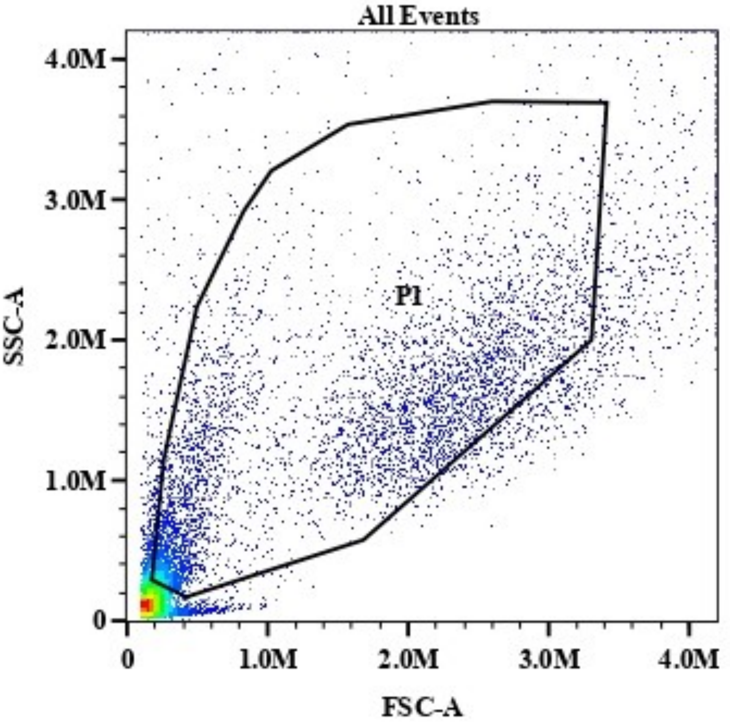


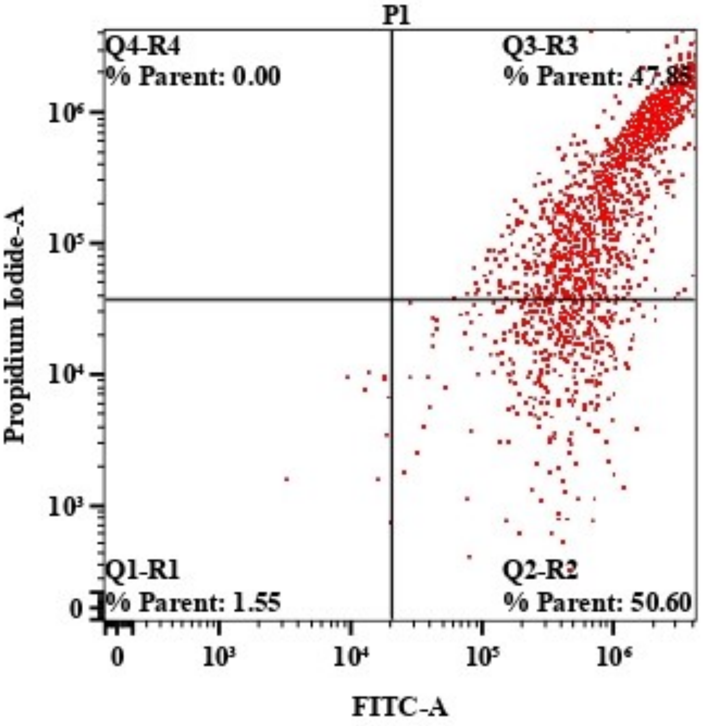


| Population Hierarchy<br>Dr Moshtaaq-HEPG2-PLANT EXTRACTION | | |
|---|---|---|
| Population | % Parent | Count |
| All Events | 100.00 | 12,248 |
| P1 | 41.66 | 5,103 |
| Q4-R4 | 0.00 | 0 |
| Q3-R3 | 47.85 | 2,442 |
| Q1-R1 | 1.55 | 79 |
| Q2-R2 | 50.60 | 2,582 |

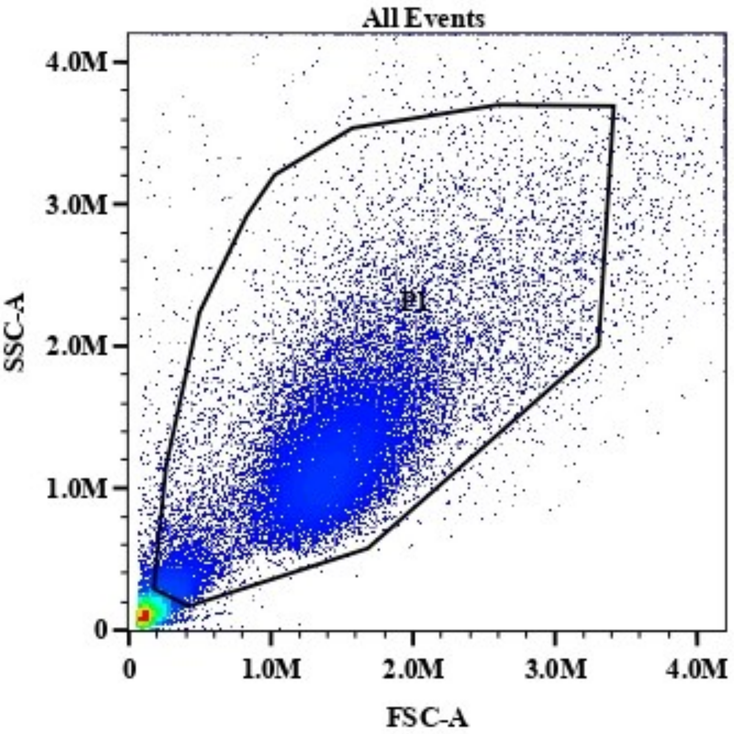


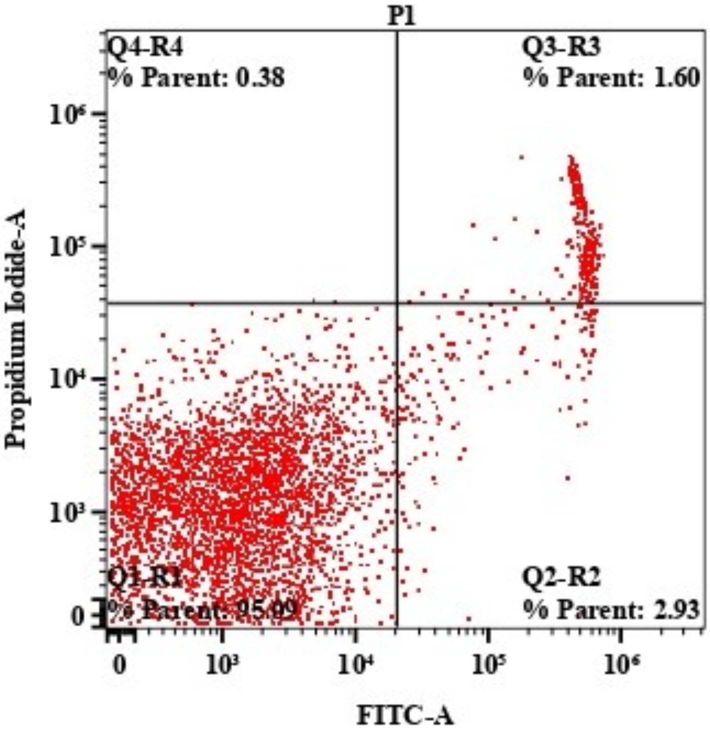


| Population Hierarchy<br>Dr Moshtaaq-HEPG2-HEPG-2 CONROL | | |
|---|---|---|
| Population | % Parent | Count |
| All Events | 100.00 | 50,000 |
| P1 | 51.99 | 25,993 |
| Q4-R4 | 0.38 | 99 |
| Q3-R3 | 1.60 | 415 |
| Q1-R1 | 95.09 | 24,717 |
| Q2-R2 | 2.93 | 762 |

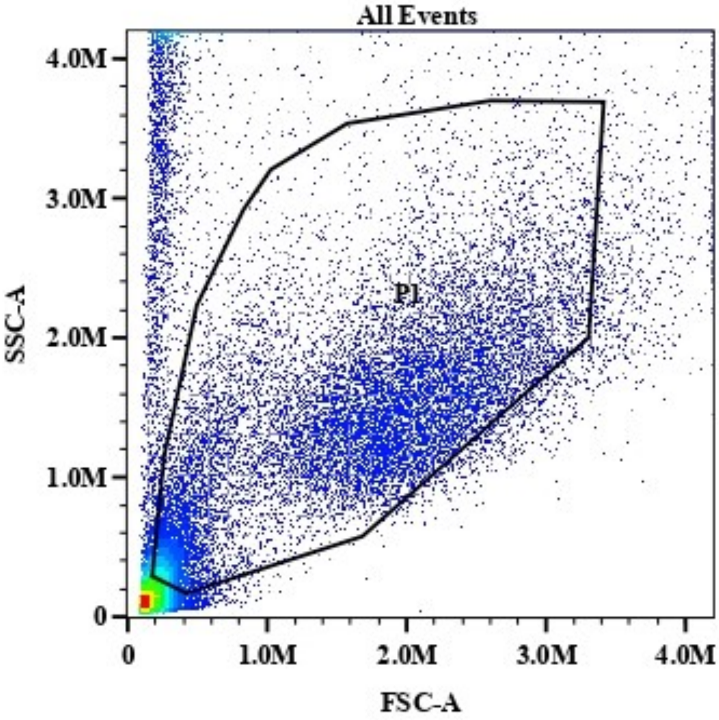


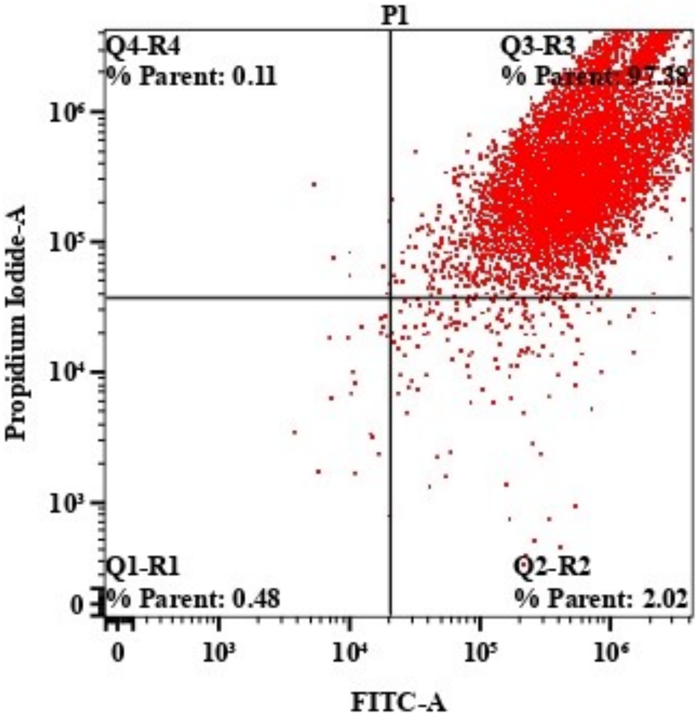


**Population Hierarchy**
**Dr Moshtaaq-HELA CELLS-PLANT EXTRACTION**

| Population | % Parent | Count |
|---|---|---|
| All Events | 100.00 | 50,000 |
| P1 | 31.81 | 15,904 |
| Q4-R4 | 0.11 | 17 |
| Q3-R3 | 97.38 | 15,488 |
| Q1-R1 | 0.48 | 77 |
| Q2-R2 | 2.02 | 322 |

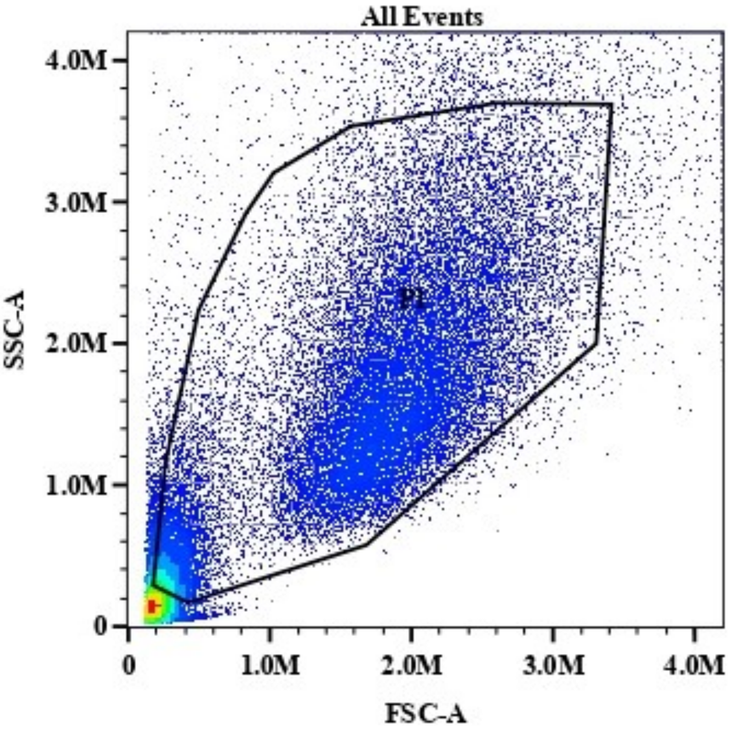


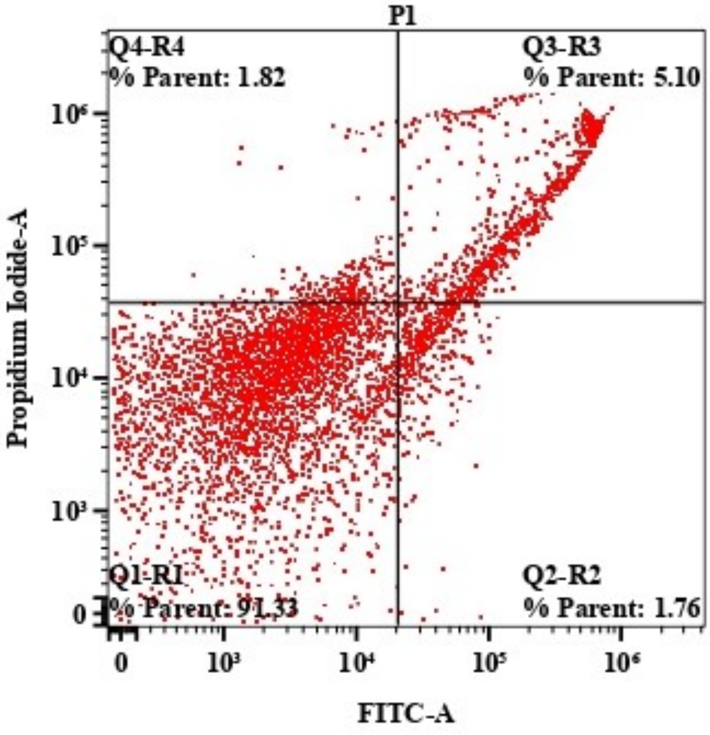


Population Hierarchy
Dr Moshtaaq-HELA CELLS-HELA CONTROL

| Population | % Parent | Count |
|---|---|---|
| All Events | 100.00 | 50,000 |
| P1 | 61.10 | 30,552 |
| Q4-R4 | 1.82 | 556 |
| Q3-R3 | 5.10 | 1,557 |
| Q1-R1 | 91.33 | 27,902 |
| Q2-R2 | 1.76 | 537 |

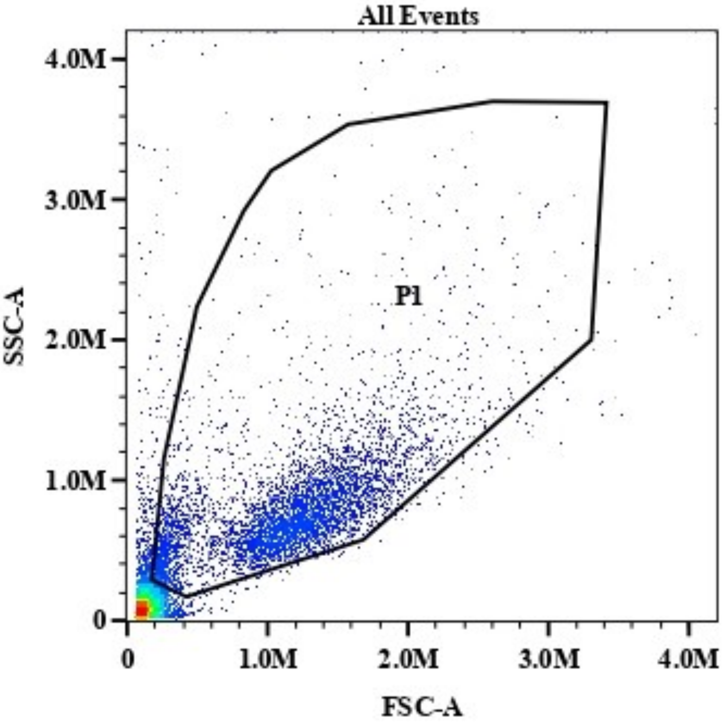


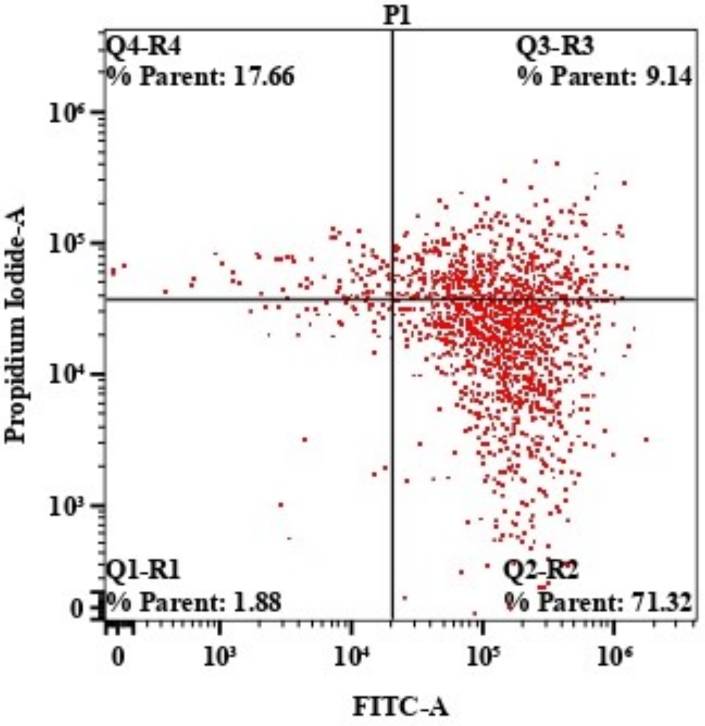


**Population Hierarchy**
**Dr Moshtaaq-HCT 116-PLANT EXTRACTION**

| Population | % Parent | Count |
|---|---|---|
| All Events | 100.00 | 12,160 |
| P1 | 42.01 | 5,108 |
| Q4-R4 | 17.66 | 902 |
| Q3-R3 | 9.14 | 467 |
| Q1-R1 | 1.88 | 96 |
| Q2-R2 | 71.32 | 3,643 |

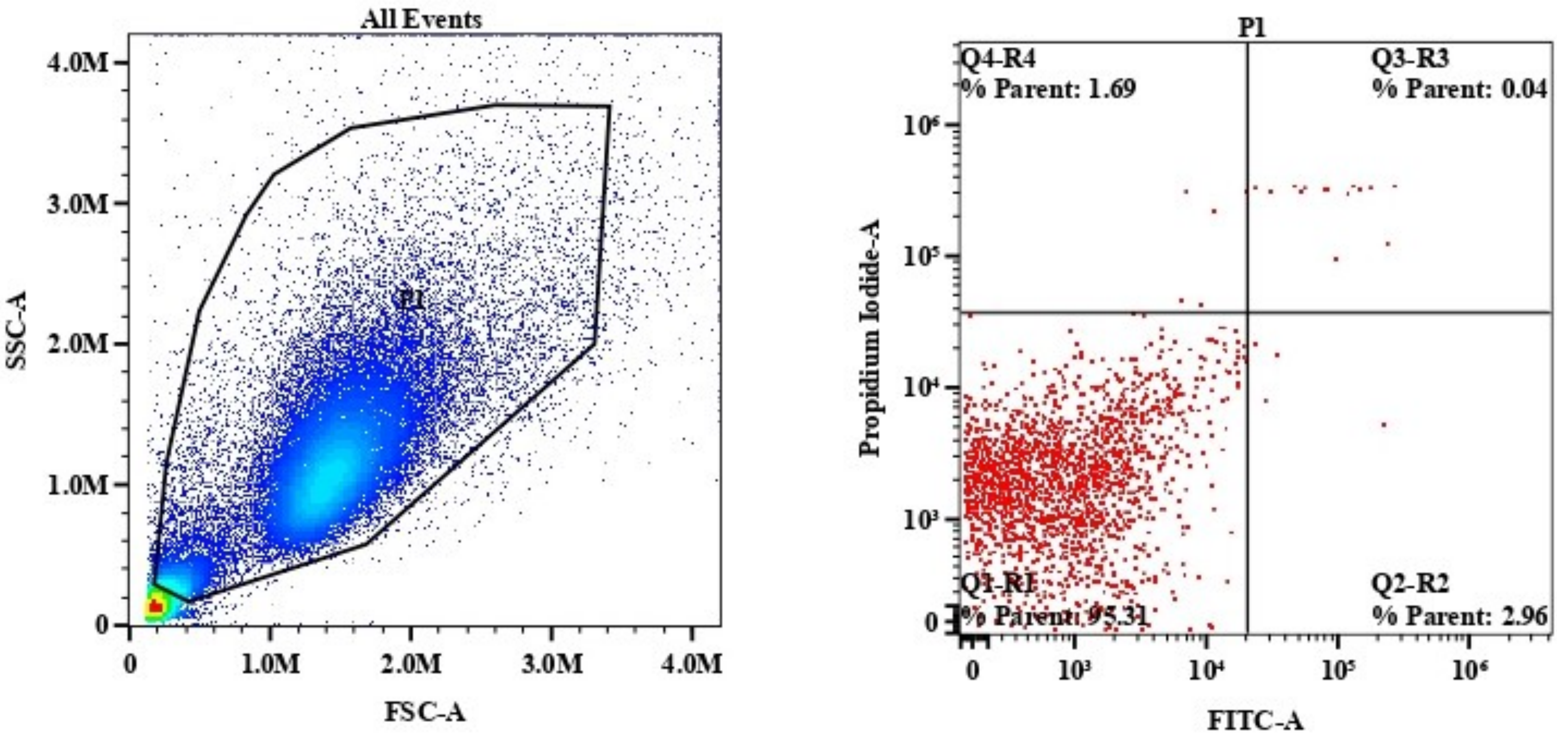


**Population Hierarchy**
**Dr Moshtaaq-HCT 116-HCT 116 CONTROL**

| Population | % Parent | Count |
|---|---|---|
| All Events | 100.00 | 50,000 |
| P1 | 73.63 | 36,817 |
| Q4-R4 | 1.69 | 622 |
| Q3-R3 | 0.04 | 14 |
| Q1-R1 | 95.31 | 35,091 |
| Q2-R2 | 2.96 | 1,090 |

Supplementary Figure S3. Distribution of cells in different phases of cell death after treatment with leaf extract of M. communis: The cells shown in the closed polygon (PI) of the left panel are the fraction of total cells gated into the channels for their enumeration into live, early apoptotic, late apoptotic, and necrotic cells (right panel). The table shows the gated cells (PI) of total cells in terms of events and percentage, and their further distribution into live (Q1-R1), early apoptotic (Q2-R2), late apoptotic (Q3-R3) and necrotic (Q4-R4) cells. For each cell line, treatment (leaf extract-treated) and control (DMSO-treated) data sets are shown.
