# Supplementary material for: Antimycobacterial and Anticancer Properties of Myrtus communis Leaf Extract
Source: Pharmaceuticals (Basel). 2024 Jul 2;17(7):872. doi: 10.3390/ph17070872 (PMC11279575; doi:10.3390/ph17070872)
Supplement: Supplementary file 1 [file pharmaceuticals-17-00872-s001.zip › pharmaceuticals-3003337-supplementary/Pharmaceuticals sup figures/Sup. Fig. S4.pdf]

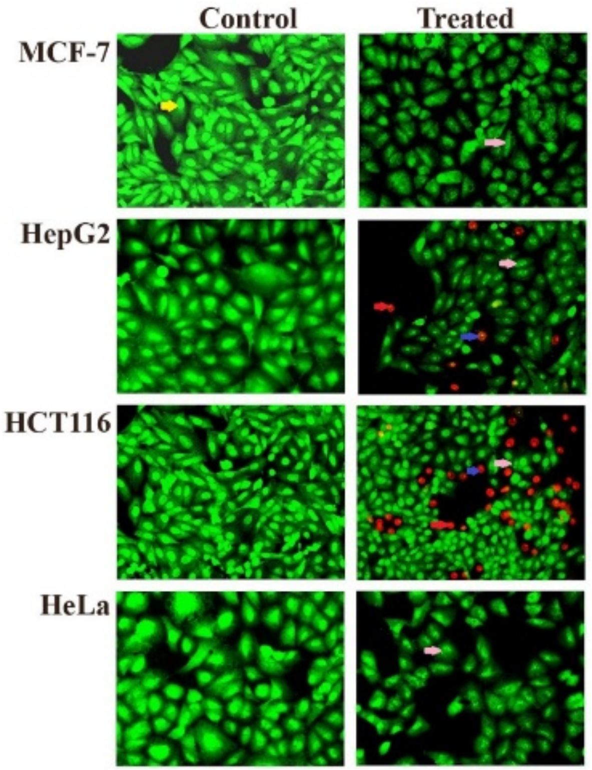

Supplementary Figure S4. M. communis leaf extract induces apoptosis. The representative fluorescent images of control and M. communis leaf extract-treated cancer cell lines MCF-7, HepG2, HCT116, and HeLa. The cells were treated for 48 hours and stained with AO/EB. Yellow arrow: live cell, pink arrow: early apoptotic, red arrow: necrotic, and blue arrow: late apoptotic. Scale bar: 20 μm.
