# Supplementary material for: Antimycobacterial and Anticancer Properties of Myrtus communis Leaf Extract
Source: Pharmaceuticals (Basel). 2024 Jul 2;17(7):872. doi: 10.3390/ph17070872 (PMC11279575; doi:10.3390/ph17070872)
Supplement: Supplementary file 1 [file pharmaceuticals-17-00872-s001.zip › pharmaceuticals-3003337-supplementary/Pharmaceuticals sup figures/Sup. Table S1.pdf]

Supplementary Table S1. Relative percentage (% peak area) of the compounds detected in ethanolic leaf extract of *M. communis* by GC-MS

S. No	RT (min)*	Compound Name	SI**	% Peak Area***
1	50.5	1,1,8a-Trimethyloctahydro-2,6-naphthalenedione	67	27.6
2	40.4	Pyrogallol	92	9.11
3	16.9	1, 8-Cineole	95	3.96
4	21.3	Linalool	97	2.8
5	7.3	Ethyl orthoformate	85	1.99
6	31.6	Linalyl formate	93	1.93
7	116.1	α-tocopherol-β-D-mannoside	96	1.78
8	30.5	5-Hydroxymethylfurfural	93	1.62
9	27.1	α-Terpineol	98	1.6
10	121.4	Clionasterol	84	1.35
11	106.3	Squalene	94	1.15
12	27.4	L-α-Terpineol	96	1.12
13	38	α-Terpinyl acetate	92	1.02
14	9.9	Dihydroxyacetone	93	1.01
15	31.9	Linalyl acetate	91	0.97
16	64.3	3-Methyl-2-butenoic acid, undec-2-enyl ester	82	0.81
17	115.1	n-Dotriacontane	95	0.72
18	72	L-Ascorbyl 2,6-Dipalmitate	91	0.66
19	16.7	D-Limonene	93	0.65
20	5.1	Acetol	98	0.64
21	6.9	Methyl pyruvate	93	0.57
22	42.2	β-Caryophyllene	94	0.56
23	6.2	Methyl acrylate	91	0.5
24	24.2	4H-Pyran-4-one, 2,3-dihydro-3,5-dihydroxy-6-methyl-	90	0.49
25	28.5	Catechol	96	0.49
26	65.8	Phytol acetate	91	0.42
27	9.5	Isopropyl isopropoxyacetate	80	0.36

28	11.4	1,2-Cyclopentanedione	97	0.32
29	79.9	9-Octadecenoic acid	88	0.3
30	14	2,4-Dihydroxy-2,5-dimethyl-3(2H)-furanone	81	0.25
31	48	Cyclohexanecarboxaldehyde, 6-methyl-3-(1-methylethyl)-2-oxo-1-(3-oxobutyl)-	81	0.25
32	10.5	Ethyl diethoxyacetate	82	0.23
33	94.9	Palmitic acid- β-monoglyceride	87	0.22
34	42.7	α -Isomethyl ionone	82	0.21
35	6.5	Methyl acetate	91	0.19
36	78.8	Phytol	85	0.19
37	20.4	(+)-4-Carene	90	0.18
38	8.7	3-Hydroxymethylfuran	94	0.17
39	20.3	5-Hydroxyazouracil	84	0.17
40	14.6	5-Diethoxymethyl-3-ethoxy-4,5-dihydro-isoxazole	80	0.14
41	6.7	Ethyl glycolate	96	0.13
42	14.1	5-Diethoxymethyl-3-ethoxy-4,5-dihydro-isoxazole	83	0.12
43	41.7	Methyleugenol	92	0.12
44	43.5	Tyrosol	94	0.11
45	13.3	5-MethyIfurfural	96	0.1
46	14.8	Glutaconic anhydride	91	0.09
47	71.7	Aspidinol	91	0.08
48	13.8	(−)-β-Pinene	95	0.07
49	15.1	2,2-Diethyl-3-methyl-1,3-oxazolidine	80	0.06
50	14.4	Phenol	90	0.04

*Rt: Retention time

**SI (Similarity Index): It is the percentage of computer-based spectral matching between the mass spectrums of each unknown peak in the chromatogram to the spectra stored in NIST library database. SI = 100; when two spectra are identical and 0 if they are completely different

***% Peak Area: It is the relative percentage amount of each component in the extract calculated by comparing its peak area to the total peak areas in the chromatogram. The table taken from Mir, M.A., Bashir, N., Alfaify, A., Oteef, M.D.Y., BMC Complement. Med. Ther. 2020, 20(86), has been reorganized with increasing order of % peak area.
